# Identification of the patient with enlarged prostate: diagnosis and guidelines for management

**DOI:** 10.1186/1750-4732-1-11

**Published:** 2007-07-09

**Authors:** Steven A Kaplan

**Affiliations:** 1 Professor of Urology and Chief, Institute for Bladder and Prostate Health, Weill Medical College of Cornell University, F9 – West, Box 261, 1300 York Avenue, New York, NY 10021, USA

## Abstract

Benign enlargement of the prostate, also referred to as benign prostatic hyperplasia, is a common condition in men. Because enlarged prostate (EP) was viewed historically as a symptomatic condition, management of voiding symptoms with α-blockers was often the goal of therapy. However, it is now recognized that EP is a progressive disorder, which may be complicated by acute urinary retention and which may eventually require EP-related surgery. The 5α-reductase inhibitors decrease dihydrotestosterone levels, which slow disease progression by causing regression of the prostate epithelial cells. These agents are considered disease modifying, and they may reduce the progression of prostate enlargement. This article reviews evaluation, diagnosis, and treatment strategies for EP, and it provides a practical algorithm for management of patients with EP.

## Background

### Review

Benign enlargement of the prostate, also referred to as benign prostatic hyperplasia (BPH), is a common occurrence in aging men. During the past decade, the role of primary care physicians (PCPs) in managing enlarged prostate (EP) has increased considerably. This is largely because men who suffer from bothersome symptoms associated with EP typically present to their PCPs for initial treatment. Data from the National Institutes of Health suggest that at least 6.3 million men in the United States aged between 50 and 79 years may be affected by EP, accounting for 4.5 million doctor visits with "hyperplasia of the prostate" as the primary diagnosis [1]. Although diagnostic and treatment recommendations are available for urologists [2], no guidelines have been specifically designed to guide PCPs in the diagnosis and management of EP. A survey found that two thirds of PCPs have only rarely or never used the American Urological Association Symptom Index (AUA-SI) – an index that provides a valid measure of a patient's symptom severity over time (based on 7 questions scored on a 0–4 scale) – when diagnosing EP [3]. Additionally, PCPs prescribed α-blockers more frequently than 5α-reductase inhibitors (5ARIs), even though 5ARIs have been shown to be more effective in management of disease progression of the prostate over time.

Because EP was viewed historically as a symptomatic condition, management of voiding symptoms was often the goal of therapy, and as such, α-blockers were advocated as primary therapy. However, today it is recognized that EP is a progressive disorder that may be complicated by acute urinary retention (AUR) and may eventually require EP-related surgery. The 5ARIs are considered disease-modifying agents because they work by decreasing dihydrotestosterone (DHT) levels, which slow disease progression by causing regression of the prostate epithelial cells. These agents also relieve voiding symptoms, improve peak urinary flow rate, and decrease risk of complications associated with EP [4,5]. The AUA guidelines recommend use of 5ARIs to prevent disease progression in men with EP [2]. Clinicians need to be aware of current treatment recommendations to appropriately manage patients with EP, thus creating the need to disseminate practical guidance tools. This article reviews evaluation, diagnosis, and treatment strategies for EP, and it provides an algorithm for management of patients with EP.

### Identification of the patient with enlarged prostate

Enlargement of the prostate becomes more common as men age, occurring in more than half of those aged between 50 and 60 years. Other risk factors that have been reported for enlarged prostate include nationality and marital status. Clinical manifestations of EP range from various degrees of lower urinary tract symptoms (LUTS) to AUR and renal failure. Clinically, patients are usually identified by the presence of LUTS, by prostate enlargement found on digital rectal examination (DRE), or by elevated prostate-specific antigen (PSA) measurement during a routine examination.

Because patients are often embarrassed to discuss prostate symptoms with physicians, LUTS may persist for years before individuals seek consultation. Men also may consider changes in urinary function to be a normal part of the aging process, or they may be reluctant to discuss symptoms because of a fear of EP-related surgery. Additionally, LUTS are not specific to EP (Table 1). Differential diagnosis of LUTS may include other urologic and nonurologic conditions, medications that increase obstructive urinary symptoms, obesity, cigarette smoking, regular alcohol consumption, and elevated blood pressure [6,7]. Thus, differential diagnoses must be critically evaluated when examining patients with LUTS.

**Table 1:** Conditions potentially associated with lower urinary tract symptoms in men [6,7]

**Urologic and nonurologic conditions**
• Prostate cancer
• Prostatitis
• Bladder cancer
• Bladder stones
• Overactive bladder
• Interstitial cystitis
• Diabetes mellitus
• Parkinson's disease
• Congestive heart failure
• Lumbosacral disc disease
• Multiple sclerosis
• Spinal cord injury
• Stroke
**Medications**
• Tricylic antidepressants
• Anticholinergic agents
• Diuretics
• Narcotics
• First-generation antihistamines
• Decongestants

A comprehensive evaluation is necessary to confirm a diagnosis of EP. The AUA guidelines recommend a careful medical history, symptom assessment using the AUA-SI score or the BPH-impact index, physical examination, urinalysis, and subsequent serum PSA test in appropriate patients to rule out cancer [2]. Although initial evaluation does not include routine serum creatinine monitoring, this measurement may be useful to exclude other causes of renal insufficiency. Presence of "alarm symptoms," such as occurrence of EP in men aged 45 years or younger, refractory retention, persistent gross hematuria, bladder stones, renal insufficiency, abnormally high PSA levels, and recurrent urinary tract infections (UTIs), may require more immediate management [8].

Prostate size should be evaluated when deciding if and how to treat. Accurately estimating prostate size and volume may be challenging with DRE, particularly in men with a larger prostate [9]. Underestimation of prostate size could have important ramifications, including inappropriate management and disease progression. As a general guideline, greater than 2-finger widths (or 2.5 for smaller fingers) diameter would indicate EP (prostate volume > 30 mL) [8].

### Assessing symptoms

Patient perception of bothersome symptoms is an important consideration in management of EP [2]. Data accumulated over the past decade clearly show that EP is a progressive disorder, which may be associated with increasingly bothersome symptoms affecting quality of life (QOL) [10]. Progression indicators may include deterioration in LUTS, increased prostate size, reduced urinary flow rate, bladder complications, hematuria, and increased AUR risk. Although AUR is not life threatening, it is a serious QOL issue for patients [11]. Consequently, prevention of AUR is desirable for men with EP, particularly those with risk factors such as moderate to severe LUTS and poor urinary-flow rates [12].

An epidemiologic study evaluated progression of BPH in over 2000 men aged 40 years or older [10]. After a 6-year, longitudinal follow-up, median peak urinary-flow rate decreased 2.1% each year. Peak urinary-flow rate declined more rapidly in men with increasing baseline age, prostate volume, and symptom severity. The largest decline in peak urinary-flow rate occurred in men aged 70 years or older and in those with a baseline rate of 10 mL/sec. Additionally, there was a slow but progressive increase in urinary symptom severity during 42 months of follow-up [13], with the greatest increases in symptom severity in men older than 60 years of age as compared with men in their 40 s. Symptom progression was evident because 14% and 22% of men who had mild symptoms at baseline reported moderate to severe symptoms after 18 and 42 months, respectively. Among men with none to mild symptoms at baseline, AUR incidence increased from 2.6/1000 person-years among men aged 40 to 49 years to 9.3/1000 person-years among men aged 70 to 79 years. In those who had moderate to severe symptoms at baseline, AUR incidence increased substantially from 3.0/1000 person-years in men aged 40 to 49 years to 34.7/1000 person-years in men aged 70 to 79 years. Additionally, men with decreased urinary-flow rate (< 12 mL/sec) at baseline had a 4-times increased risk for AUR compared with those with higher urinary flow rates (> 12 mL/sec). Men with a prostate larger than 30 mL at baseline had a 3-times increased AUR risk. Therefore, disease progression is clearly associated with increased AUR risk.

**Figure 1:**
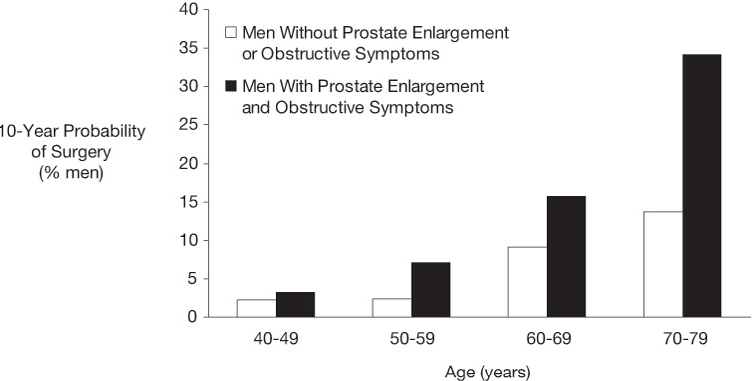
**Prostate enlargement contributes to risk of BPH-related surgery [14]**. BPH = benign prostatic hyperplasia.

Prevention of AUR progression is desirable because prostatectomy resulting from AUR is associated with increased morbidity and mortality [12]. In the Baltimore Longitudinal Study of Aging, men with EP and obstructive symptoms were up to 8 times more likely to require prostatectomy within 10 years than men of the same age without EP [14]. Advancing age also increased the 10-year probability for prostatectomy (Figure 1).  The 10-year probability for EP-related surgery was highest (34%) in men aged 70 years or older with EP and obstructive symptoms. Progression of AUR and the "threat" of eventual EP-related surgery is a major concern for patients. A survey conducted in Canada found that nearly 60% of men with EP were concerned about developing AUR, and that nearly 70% were significantly concerned about the need for EP-related surgery [11]. Catheter insertion for AUR also was considered extremely detrimental to QOL.

### Medical management of the enlarged prostate: disease modification

**Figure 2:**
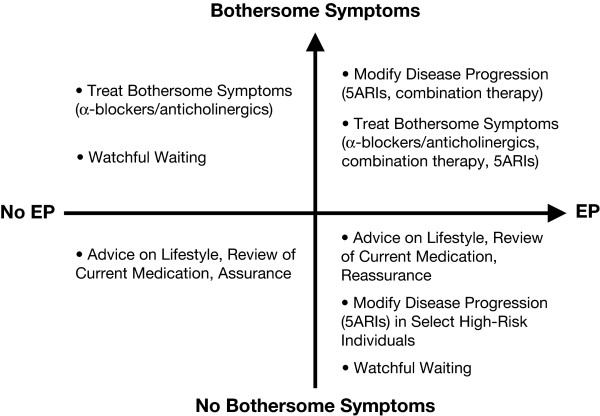
**Goals of therapy for patients diagnosed with enlarged prostate**. (adapted from reference 8, with permission). 5 ARIs = 5α-reductase inhibitor; EP = enlarged prostate.

The goals of EP management should include disease modification and treatment of bothersome symptoms. Depending on size of prostate and symptoms, treatment options may include watchful waiting (ie, patient monitored by physician with no active intervention), symptom management, or disease modification (Figure 2).

In the past, treatment primarily focused on symptom management with anticholinergic agents or α-blockers. Patients who suffer from urge incontinence resulting from overactive bladder may be treated with anticholinergic agents (eg, oxybutynin, tolterodine, solifenacin). For patients with obstructive symptoms resulting from EP, α-blockers (eg, terazosin, doxazosin, tamsulosin, alfuzosin) could improve urinary flow rates. The α-blockers target smooth muscle receptors in the prostate and bladder neck by relaxing muscle fibers, and thus making it easier to urinate. All α-blockers are similarly efficacious at improving urinary flow rates by 15% to 30% although tolerability profiles may differ among agents [15]; adverse effects may include orthostatic hypertension, dizziness, and ejaculatory problems. Anticholinergics and α-blockers are considered symptom modifying because they do not affect the natural course of the disease.

Unlike symptom-modifying agents, 5 ARIs inhibit the 5α-reductase enzymes responsible for the conversion of testosterone to DHT. The 5 ARIs decrease DHT levels, slowing the disease process and causing shrinkage of prostate epithelial cells. 5 ARI therapy is considered disease modifying because it treats the underlying cause of the disease. Dutasteride and finasteride are the 5 ARIs currently available in the United States, and both agents effectively relieve symptoms and prevent disease progression in men with EP. Dutasteride inhibits both type-1 and type-2 isoforms of the 5α-reductase enzyme, resulting in greater than 90% suppression of DHT [16], whereas finasteride only inhibits the type-2 isoenzyme, reducing DHT levels by 45% to 85% [17]. Even though adverse effects on sexual function (eg, impotence, decreased libido) have been reported by patients on 5 ARIs, they are typically reversible and, after 6 months, no more common than with placebo [18].

The landmark Medical Therapy of Prostatic Symptoms (MTOPS) study showed that treatment with a 5 ARI significantly reduced the rate of AUR and prevented progression of EP [4]. In this 4.5-year, double-blind, placebo-controlled trial, 3047 men with symptomatic BPH were randomized to receive doxazosin or finasteride, alone or in combination, to evaluate delay or prevention of clinical progression of BPH. Overall clinical progression, defined by a composite end point of a 4-point or greater increase in AUA-SI score above baseline, AUR, urinary incontinence, renal insufficiency, or recurrent UTI, was 17% with placebo, 10% with doxazosin (*P *< .001), 10% with finasteride (*P *= .002), and 5% with the combination (*P *< .001). The rate of AUR was significantly lower in both the finasteride (68% risk reduction; *P *= .009) and the combination groups (81% risk reduction; *P *< .001) compared with placebo. Finasteride (64%) and combination therapy (67%) significantly reduced risk of invasive therapy (*P *< .001 compared with placebo for both groups). Although doxazosin delayed time to AUR, it did not significantly reduce the cumulative incidence of AUR or invasive therapy, when compared with placebo.

In an evaluation of 4325 men with clinical BPH with moderate to severe symptoms, treatment with dutasteride for 24 months reduced serum DHT from baseline by a mean of 90.2% (*P *< .001 compared with placebo) [5]. Compared with placebo, dutasteride reduced total prostate volume from baseline by 25.7% at 24 months, transition zone volume by 20.4%, and symptom score by 4.5 points, and it increased maximal flow rate by 2.2 mL/s (*P *< .001). The efficacy of dutasteride was further evaluated in a 2-year, open-label extension of 3 phase III randomized studies [19]. After 48 months, the dutasteride/dutasteride combination group experienced improvement in symptoms (AUA-SI score decreased 6.5 points;*P *< .001), increased urine flow by 2.7 mL/s (Q_max_; *P *= .042), and reduced prostate volume by 27.3% (*P *< .001) from baseline compared with the placebo/dutasteride group. In the Proscar Long-term Efficacy and Safety Study, which evaluated the efficacy of finasteride compared with placebo in 3040 patients with BPH, prostate volume decreased by 18% in the finasteride-treated patients compared with a 14% increase in the placebo group [20]. Patients receiving finasteride also experienced improvement in voiding symptoms and peak urinary flow rate compared with placebo. These findings support the AUA recommendation of using 5 ARIs for long-term disease management in patients with LUTS and with evidence of EP [2].

### Treatment guidelines

**Figure 3:**
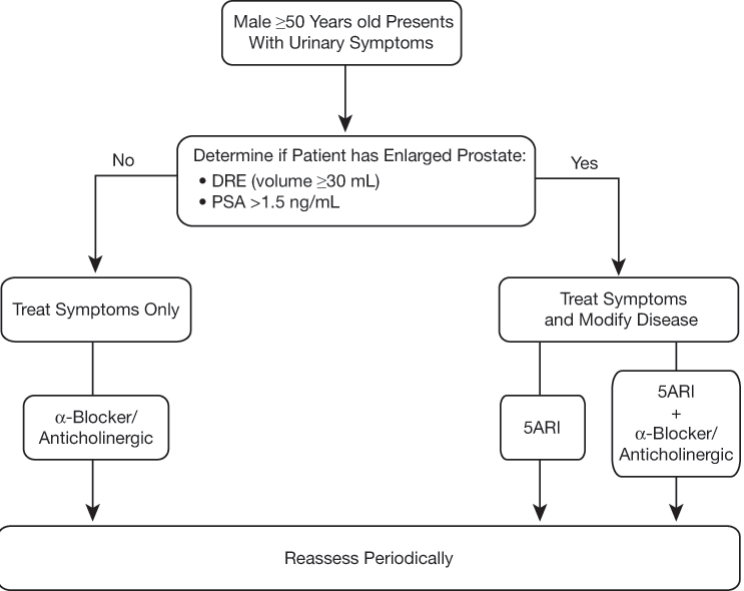
**Practical algorithm for management of patients with enlarged prostate associated with bothersome symptoms [8]**. 5 ARI = 5α-reductase inhibitor; DRE = digital rectal exam; PSA = prostate-specific antigen.

Figure 3 provides a practical algorithm for treatment of EP. For men with a smaller prostate (< 30 mL) and no bothersome symptoms, advice on lifestyle modifications (eg, weight loss, restricting fluids at night), review of current medications, and watchful waiting are recommended. The AUA guidelines advocate watchful waiting for patients with mild (AUA-SI ≤ 7), moderate, or severe symptoms (AUA-SI ≥ 8) who are not bothered by their symptoms [2]. For men with a smaller prostate (< 30 mL) who experience bothersome symptoms, symptomatic treatment with α-blockers (and/or anticholinergics) may be beneficial. Vasodilatory adverse events, such as dizziness, should be monitored when initiating α-blocker therapy.

For men with EP (≥ 30 mL) and no bothersome symptoms, the AUA guidelines recommend use of 5 ARIs to prevent disease progression, although watchful waiting may be considered [2]. For men with EP (≥ 30 mL) and bothersome symptoms, a 5 ARI should be used to shrink the prostate, thereby improving urinary symptoms, and reducing AUR risk and the need for subsequent EP-related surgery. For the long term, some of these patients may be managed with a 5 ARI alone. An α-blocker (and/or anticholinergics) may be prescribed for patients who experience particularly bothersome symptoms. Combination therapy with an α-blocker (for early symptom reduction) and 5 ARI (for long-term disease management) is most appropriate in men who are symptomatic and who have a high risk for progression (ie, enlarged prostate, age > 70 years, high symptom score) [2]. For patients on combination therapy, the αblocker may be discontinued in the majority of men once the therapeutic efficacy of the 5 ARI is confirmed (typically within 6 to 9 months) [21,22] although some individuals may benefit from continuation of combination therapy. Even though the AUA guidelines do not provide specific recommendations for follow-up, patients undergoing treatment for EP should be periodically assessed for disease progression [8]. Urologic referral may be indicated for patients with disease progression while on 5 ARI therapy, men with suspected malignancy based on elevated PSA or physical examination, or individuals experiencing gross hematuria.

## Conclusion

Benign enlargement of the prostate is a common condition. As the disease progresses, LUTS increase and associated complications become more common. Current guidelines for management of EP emphasize the use of 5 ARIs either alone or in combination with an α-blocker. The use of α-blockers afford symptom relief, but they do not affect disease progression. The 5 ARIs provide symptomatic relief, reduce prostate volume, and slow the disease process by shrinking prostate epithelial cells. Initiation of 5 ARI therapy in symptomatic men with EP is a reasonable approach to disease management, which may prevent long-term negative consequences.

## Competing interests

GlaxoSmithKline: consultant

National Institutes of Health: investigator

Pfizer Inc: investigator, consultant

Sanofi: consultant

Astellas: investigator, consultant

## Authors' contributions

SK was involved in the conception, drafting, revising, and final approval of the important intellectual content for this manuscript
